# Maize Crop Coefficient Estimated from UAV-Measured Multispectral Vegetation Indices

**DOI:** 10.3390/s19235250

**Published:** 2019-11-29

**Authors:** Yu Zhang, Wenting Han, Xiaotao Niu, Guang Li

**Affiliations:** 1 Institute of Soil and Water Conservation, Chinese Academy of Sciences and Ministry of Water Resources, Yangling 712100, China; zhangyu16@mails.ucas.ac.cn (Y.Z.); niuxiaotao16@mails.ucas.ac.cn (X.N.); 2 University of Chinese Academy of Sciences, Beijing 100049, China; 3 Key Laboratory of Agricultural Internet of Things, Ministry of Agriculture, Yangling 712100, China; 4 Institute of Soil and Water Conservation, Northwest A&F University, Yangling 712100, China; 5 College of Mechanical and Electronic Engineering, Northwest A&F University, Yangling 712100, China; liguangai100@163.com

**Keywords:** crop coefficient (*K_c_*), vegetation indices, deficit irrigation, regression model, soil water balance, stress coefficient

## Abstract

The rapid, accurate, and real-time estimation of crop coefficients at the farm scale is one of the key prerequisites in precision agricultural water management. This study aimed to map the maize crop coefficient (*K_c_*) with improved accuracy under different levels of deficit irrigation. The proposed method for estimating the *K_c_* is based on multispectral images of high spatial resolution taken using an unmanned aerial vehicle (UAV). The analysis was performed on five experimental plots using *K_c_* values measured from the daily soil water balance in Ordos, Inner Mongolia, China. To accurately estimate the *K_c_*, the fraction of vegetation cover (*f_c_*) derived from the normalized difference vegetation index (*NDVI*) was used to compare with field measurements, and the stress coefficients (*K_s_*) calculated from two vegetation index (VI) regression models were compared. The results showed that the *NDVI* values under different levels of deficit irrigation had no significant difference in the reproductive stage but changed significantly in the maturation stage, with a decrease of 0.09 with 72% water applied difference. The *f_c_* calculated from the *NDVI* had a high correlation with field measurement data, with a coefficient of determination (R^2^) of 0.93. The ratios of transformed chlorophyll absorption in reflectance index (*TCARI*) to renormalized difference vegetation index (*RDVI*) and *TCARI* to soil-adjusted vegetation index (*SAVI*) were used, respectively, to establish two types of *K_s_* regression models to retrieve *K_c_*. Compared to the *TCARI*/*SAVI* model, the *TCARI*/*RDVI* model under different levels of deficit irrigation had better correlation with *K_c_*, with R^2^ and root-mean-square error (RMSE) values ranging from 0.68 to 0.80 and from 0.140 to 0.232, respectively. Compared to *K_c_* calculated from on-site measurements, the *K_c_* values retrieved from the VI regression models established in this study had greater ability to assess the field variability of soil and crops. Overall, use of the UAV-measured multispectral vegetation index approach could improve water management at the farm scale.

## 1. Introduction

Water scarcity is a major factor limiting irrigated agriculture, especially in arid and semi-arid areas of the world. Due to global climate changes and the imbalance between water supply and demand, it is particularly necessary to improve crop water use [1]. Therefore, the regulated deficit irrigation (RDI) strategy for reducing water consumption is widely used in current agriculture [2]. In this context, how to accurately estimate and monitor crop water requirements is not only the key for optimizing irrigation scheduling and improving water use efficiency, but also essential research for enhancing food production and *SAVI*ng regional water resources [3,4].

A common indicator of crop water requirements is the crop coefficient (*K_c_*), which was presented in FAO 56 [5] and is used to estimate crop evapotranspiration (*ET_c_*) by multiplying the reference crop evapotranspiration (ET_0_) [6]. The *K_c_* primarily depends on meteorological information, crop-specific coefficients, the lengths of crop growth stages, and plant-available soil water [7]. It can be estimated using single and dual *K_c_* approaches. The single approach is the averaging of *K_c_* trends that incorporate plant transpiration (*K_cb_*) and soil evaporation (*K_e_*). Compared to the single approach, the dual approach improves the estimation accuracy of ET by considering the plant transpiration and soil evaporation separately, i.e., *K_c_* = *K_cb_* + *K_e_* [8]. However, in practical crop conditions, the *K_c_* needs to be appropriately adjusted by using a stress coefficient (*K_s_*) for nonstandard conditions, especially water stress conditions [6]. The dual approach has been appropriately used for crops at a field or regional scale [9,10,11,12,13].

To date, several methods have been presented to monitor *K_c_*, such as soil water balance, eddy covariance, Bowen ratio, lysimeter, and remote sensing [14,15,16,17,18]. In situ measurements are likely to be time-consuming and costly, and make it hard to consider the spatial variability of crops and soil [19]. Therefore, remote sensing of *K_c_* has become increasingly recommended in irrigation management. One of the most common approaches is to estimate the real-time *K_cb_* and *K_c_* through empirical equations of vegetation indices (VIs) such as the normalized difference vegetation index (*NDVI*) derived from multispectral images [20,21,22,23]. Such empirical equations rely on the close relationship between the VIs and various actual plant growth parameters, e.g., leaf area index [24,25], fraction of ground covered by plants [26,27], and biomass [24,28]. However, Pereira et al. [7] noted that *NDVI*-based methods can not accurately observe a decrease in *K_c_* and *K_cb_* when crops are under water stress. Zhang and Zhou [29] proposed that crop water status can be accurately monitored using the VIs which are not only sensitive to water information but also contain vegetation growth status. Consequently, partial VIs such as the reflectance index (*TCARI*), optimization of soil-adjusted vegetation index (OSAVI), soil-adjusted vegetation index (*SAVI*), and renormalized difference vegetation index (*RDVI*) can effectively monitor crop water status [30]. For example, Haboudane et al. [31] utilized *TCARI*/OSAVI to predict maize water status in Canada and reported a high correlation between *TCARI*/OSAVI and chlorophyll content. Zhang et al. [19] evaluated maize water stress and its spatial variability by multispectral remote sensing. The results showed that two regression models based on *TCARI*/*RDVI* and *TCARI*/*SAVI* were able to monitor the crop water stress index (*CWSI*) with respective coefficients of determination (R^2^) of 0.81 and 0.80 under different levels of deficit irrigation.

Unmanned aerial vehicles (UAVs) in remote sensing have exponentially increased in applications of precision agriculture over the past decade [32]. Compared to conventional data acquisition methods for remote sensing, such as satellites and spectrometers [33,34], UAVs offer many obvious advantages to support water resource management and planning [35,36]. One of the most common advantages is that UAVs can provide high-quality data at the required scale and time, while conventional methods have not been applied in practice at the farm scale due to their coarse spatial resolution, infrequent coverage, and high cost [37,38]. Additionally, UAVs can be used to obtain low-cost data as frequently as necessitated by the monitoring task because of their ease of operation and deployment, their high flexibility, and the decreasing cost of the platform [39]. Thus, UAV-based monitoring has become increasingly pervasive in supporting the real-time control of irrigation systems.

In recent works, the abovementioned *K_s_* has usually been calculated using the FAO-56 soil water depletion method proposed by Allen et al. [5]. For example, Pôças et al. [6] estimated the actual *K_c_* for maize, barley, and olive based on VIs and a soil water balance model under water stress conditions. The results showed that the *K_s_* computed using the soil water balance model could accurately exhibit reductions in *K_c_* due to water stress. Another method to determine *K_s_* is based on indications of the infrared canopy temperature, such as the *CWSI*. Kullberg et al. [40] compared the performance of several canopy temperature methods converted into *K_s_*. The results showed that the *CWSI* based on infrared thermal radiometry (IRT) had the best accuracy compared to other methods, and it is typically considered to be scalable to *K_s_*. Bellvert et al. [41] stated that mapping *CWSI* from UAV thermal imagery has the limitation of inevitable mixed temperatures coming from both the soil and leaves due to bigger pixels and full cover. Considering the relationship between VIs and the *CWSI* discussed above, VIs have been found to have a comparable ability to monitor *K_c_* under water stress conditions. However, research using VIs derived from a UAV multispectral remote sensing system is still relatively rare in *K_c_* estimation at the farm scale under different levels of water stress.

Therefore, in this study, we established a *K_c_* empirical model based on UAV multispectral remote sensing to represent the crop evapotranspiration of summer maize under different levels of deficit irrigation. The main objectives were as follows:

(1) To explore the differences in *K_c_* with regard to the soil water balance in response to water stress treatments at different growth stages;

(2) To establish *K_c_* regression models based on UAV multispectral VIs that are sensitive to maize water stress and compare them with measured crop coefficients;

(3) To obtain *K_c_* maps derived from the *K_c_* regression model with high spatial–temporal resolution at the farm scale.

## 2. Materials and Methods

### 2.1. Study Area

The study was carried out on an experimental farm located in Zhaojun Town, southwest Inner Mongolia, China (40°26′0.29” N, 109°36′25.99” E). The experimental area is approximately 1.13 ha at 1010 m altitude above the sea level. The climate is semi-arid, and the soil type is loamy sand with 80.7% sand, 13.7% powder, and 5.6% clay. The average field capacity (0–90 cm soil depth) is 0.169 m^3^·m^−3^, and the average soil bulk density is 1.56 g·cm^−3^. The soil pH, C content, and organic matter are 9.27, 27.35 g/Kg, and 47.17 g/Kg, respectively. Maize (Junkai 918) was sowed on 20 May 2017 (day of year (DOY) 140), with a 0.58 m planting distance and 0.25 m plant spacing, and the row direction was from east to west. The maize emerged on 1 June, headed on 20 July, and was harvested on 7 September (silage), giving a 110-day lifespan [19].

### 2.2. Experimental Design

**Figure 1:**
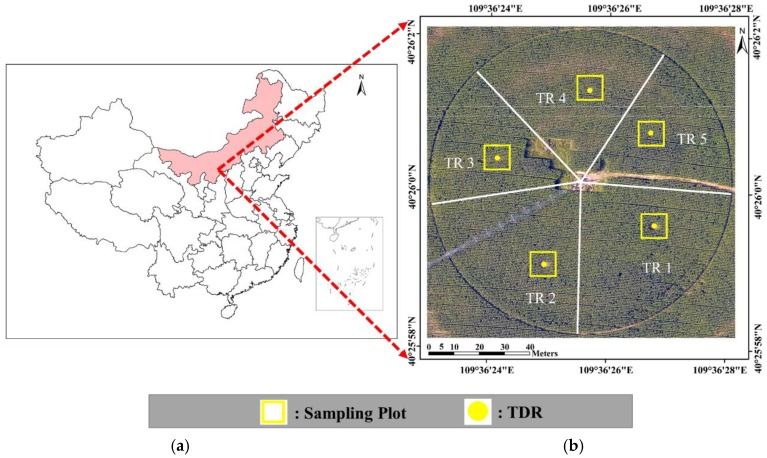
Location and region division of the research field: (**a**) location of the research field in China; (**b**) aerial view of the research field indicating treatment region division, the locations of the sampling plots, and time domain reflectometry (TDR) probes. The aerial image was taken on day of year (DOY) 185.

There were five different levels of deficit irrigation treatments for which we divided the study field into treatment (TR) regions (Figure 1b). In order to effectively collect data and control irrigation water, a 12 × 12 m^2^ experimental plot was chosen in each treatment region. The five treatments were full irrigation (TR1), slight water stress (TR5), moderate water stress (TR2 and TR3), and severe water stress (TR4). TR1 represented the total crop water requirement of fully watered maize during the whole growth period. The different levels of deficit irrigation were designed according to the percentages of applied water depth of TR1 during the late vegetation, reproductive, and maturation stages. For example, during the maturation stage, 52% of the applied water depth at TR1 was applied to TR2 (Table 1).

A central pivot sprinkler system (Valmont Industries, Inc., Omaha, NE, USA) was used for irrigation, and different irrigation amounts were processed in each treatment region by adjusting the speed of the sprinkler system. The test for water application uniformity of the central pivot irrigation system was carried out in accordance with the standards ANSI/ASAE S436.1 and ISO 11545. The uniformity coefficient for the first span (research area) of the R3000 sprinklers was calculated using the modified formula by Heermann and Hein [42], and the values were 82.7% and 88.3% under 20% and 40% of full walking speed, respectively. The amount of water applied to each treatment was measured and recorded using a MIK-2000H flow meter (Meacon Automation Technology co., Ltd., Hangzhou, China). Due to the influence of rainfall, the actual applied water depth (irrigation and rainfall amount) for each growth stage in each treatment region is shown in Table 1. In order to eliminate the stress of nutrients and weeds, fertilizer and herbicide were applied according to planting experience.

In order to obtain daily data on the soil water content, each of the five experimental plots was installed with a monitoring station with a time domain reflectometry (TDR) probe (TDR 315L, Acclima, Inc., Boise, ID, USA). The distribution of voltage pulses was done around a coaxial cable of length 3 m, and this cable was connected to a TDR 315L probe (0.15 m in length). Access tubes were installed vertically up to 90 cm into the soil in the middle of each plot, and the probe was inserted into the soil to access the tubes at different depths (30, 60, 90 cm) for the measurement of the daily volumetric soil water content (SWC) during the study period.

### 2.3. Meteorological Data

The weather data were recorded by an automated weather station located at a farm adjacent to the research field. The daily and hourly measured weather variables included rainfall, air temperature and relative humidity, net solar radiation, and wind speed (2 m above the reference grass surface). The main mean meteorological data during the study period, including the late vegetative stage (06.26–07.28), reproductive stage (07.29–08.20), and maturation stage (08.21–29), are shown in Table 2.

### 2.4. Crop Coverage Measurement

A DJI Phantom 4 Pro with an 84° field of view lens, an f/2.8 aperture, and a resolution of 4864 × 3648 pixels was used to obtain crop coverage (*f_c_*). The flights were conducted every 3 to 7 days between 11:00 and 13:00 local time, at 50 m altitude, and with a ground sample distance of 1.4 cm. The overlap of imagery to the front and side was 80%. The mosaic RGB images were acquired using Pix4DMapper software (Lausanne, Switzerland). The RGB images of each sampling plot were classified into soil and vegetation using supervised classification aided by ENVI 5.3 software. The *f_c_* value was derived as the percentage detected as vegetation.

### 2.5. Soil Water Balance

**Figure 2:**
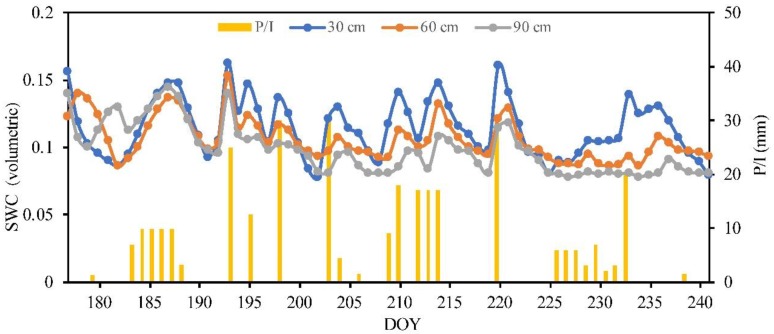
The changing curve of the average soil water content (SWC, volumetric) at the depths of 30, 60, and 90 cm during study period in 2017. The blue, orange, and gray solid lines represent the 30, 60, and 90 cm SWC, respectively. The yellow bar represents the depth of precipitation (P) and irrigation (I).

The daily crop evapotranspiration (*ET_c_*) was obtained using the soil water balance equation with soil water content measured by TDR [5,43,44,45] (Equation (1)):

(1) $$ET_{c}=P+I\pm \Delta SM-D_{p}-RO+CR$$

where *ET_c_* represents crop evapotranspiration; *P* represents the precipitation; *I* represents the irrigation; Δ*SM* represents the change in water content between two successive days, calculated by TDR; *DP* represents deep percolation; *RO* represents surface runoff; and *CR* represents capillary rise from the deep water table, which can be ignored due to the shallow to deep water table depth (3–55 m) and also due to no contribution from groundwater with capillary rise into the root zone [46]. All terms in the soil water balance are in millimeters. An example of the results of the changing curve of the average SWC (volumetric) at the depths of 30, 60, and 90 cm at TR1 during the study period is shown in Figure 2.

### 2.6. UAV Multispectral Imagery Acquisition

A multispectral camera (RedEdge, MicaSense, Inc., Seattle, WA, USA) was installed on a UAV platform (a six-rotor unmanned aircraft S900, manufactured by DJI). The S900 six-rotor UAV has the advantages of stable flight and takeoff, strong wind resistance, and low cost. The maximum take-off weight is 6 kg, the maximum payload is 2 kg, the maximum wind speed it can withstand is 5 m/s, and its flight time is 18 min. The RedEdge multispectral camera consists of five bands in the VIS–NIR spectral range at 475, 560, 668, 717, 840 nm, respectively; a 5.5 mm fixed lens; image resolution of 1280 × 960 pixels; and angle of view of 47.2° (H). The flight control board used was a Pixhawk autopilot (CUAV, Guangzhou, China), and the ground control station software Mission Planner was used to conduct the flight planning.

UAV multispectral data from fourteen flights were acquired at a 70 m flight height with 4.7 cm spatial resolution during the study period (2017.06.26~08.29) between 11:00 and 13:00 local time. The heading and side overlap and speed of the UAV were 80% and 5 m/s, respectively. The mosaic multispectral images were acquired using the photogrammetric software Pix4DMapper (Lausanne, Switzerland). In order to calibrate the multispectral images, a diffuse reflector (reflectivity 58%, size 3 × 3 m, Group VIII, Seattle, WA, USA) was used during the data collection. The measured image radiances were later converted to reflectance values to obtain spectral reflectance images.

### 2.7. The Vegetation Index Approach for Crop Coefficient Estimation

In the present study, the calculation of *K_c_* was made based on the dual crop coefficient approach. This approach divides the total crop coefficient into crop transpiration (*K_cb_*) and soil evaporation (*K_e_*) fractions [47,48]. The *K_c_* can be calculated as follows:

(2) $$K_{c}=K_{cb}+K_{e}$$

where *K_cb_* values were estimated based on *NDVI* measurements developed in a modified approach by Er-Raki et al. [49], who used 1.07 as the *K_cb,max_* value for durum wheat in a semi-arid climate in Morocco. In our study, we used 1.15 in Equation (3) as the *K_cb,max_* value for maize according to Allen et al. [5]. In addition, *K_e_* values were calculated from the fraction of vegetation cover (*f_c_*), which is strongly related to the *NDVI* [50]. Therefore, *K_cb_* and *K_e_* in the *NDVI* approach were derived as [5,51] follows:

(3) $$K_{cb}=1.15*(1-(NDVI_{max}-NDVI)/(NDVI_{max}-NDVI_{min}))$$

(4) $$K_{e}=0.9*(1-f_{c})$$

(5) $$f_{c}=1.19*(NDVI-NDVI_{min})$$

where *NDVI_max_* and *NDVI_min_* are the maximal and minimal measured *NDVI* values during the growing period. We took values of 0.88 for *NDVI_max_* and 0.14 for *NDVI_min_* according to the UAV map. The value 0.9 in Equation (4) was determined according to FAO 56 [5] based on the observed frequency of irrigation and rainfall. The value 1.19 in Equation (5) was determined according to González-Piqueras et al. [51], based on the *f_c_* being less than 80% for maize.

However, the above formulas represent potential crop evapotranspiration conditions. When water stress occurs, the stress coefficient *K_s_* should be considered. *K_s_* (0 ≤ *K_s_* ≤ 1) is defined as the ratio of actual evapotranspiration (*ET_a_*) to potential evapotranspiration (*ET_p_*), proposed by Allen et al. [5]. The *K_s_* can be calculated as follows:

(6) $$K_{s}=ET_{a}/ET_{p}=K_{c}{\;}_{act}/K_{c}.$$

Based on the principle of energy balance, Jackson et al. [52] derived a calculation model for the crop water stress index (*CWSI*) depending on the canopy temperature. The model establishes the relationship between *K_s_* and *CWSI* as

(7) $$CWSI=1-ET_{a}/ET_{p}=1-K_{s}.$$

Additionally, *CWSI* can be estimated by two VI regression models proposed by Zhang et al. [19] under different levels of deficit irrigation.

(8) $$CWSI-1=\{\begin{array}{cc} \;0 & \left(TCARI/RDVI\leq 0.195\right)\\ 2.41*(TCARI/RDVI)-0.47 & \left(0.195<TCARI/RDVI<0.609\right)\\ \;1 & \left(0.609\leq TCARI/RDVI\right) \end{array}$$

(9) $$CWSI-2=\{\begin{array}{cc} \;0 & \left(TCARI/SAVI\leq 0.182\right)\\ 2.46*(TCARI/SAVI)-0.45 & \left(0.182<TCARI/SAVI<0.589\right)\\ \;1 & \left(0.589\leq TCARI/SAVI\right) \end{array}$$

Therefore, in relation to *K_s_* and actual *K_c_* (*K_c act_*), *K_s_* and *K_c act_* are derived as follows:

(10) $$K_{c\;act}=K_{s}*K_{c}=(1-CWSI)*(K_{cb}+K_{e}).$$

Combining all the above formulas, two different models for *K_c_* estimation can be finally obtained as follows.

(11) $$K_{c}-1=\{\begin{array}{c} \;1.15*(1-(NDVI_{max}-NDVI)/(NDVI_{max}-NDVI_{min}))\\ \;+0.9*(1-1.19*(NDVI-NDVI_{min}))\;(TCARI/RDVI\leq 0.195)\\ \left(1.47-2.41*(TCARI/RDVI))*(1.15*(1-(NDVI_{max}-NDVI)/(NDVI_{max}-NDVI_{min})\right)\\ \;+0.9*(1-1.19*(NDVI-NDVI_{min})))\;(0.195<TCARI/RDVI<0.609)\\ \;0\;\left(0.609\leq TCARI/RDVI\right) \end{array}$$

(12) $$K_{c}-2=\{\begin{array}{c} \;1.15*(1-(NDVI_{max}-NDVI)/(NDVI_{max}-NDVI_{min}))\\ \;+0.9*(1-1.19*(NDVI-NDVI_{min}))\;(TCARI/SAVI\leq 0.182)\\ \left(1.45-2.46*(TCARI/SAVI))*(1.15*(1-(NDVI_{max}-NDVI)/(NDVI_{max}-NDVI_{min})\right)\\ \;+0.9*(1-1.19*(NDVI-NDVI_{min})))\;(0.182<TCARI/SAVI<0.589)\\ \;0\;(0.589\leq TCARI/SAVI) \end{array}$$

### 2.8. Vegetation Index Calculations

To establish a regression model between UAV-measured multispectral VIs and *K_c_*, *NDVI*, *TCARI*/*RDVI*, and *TCARI*/*SAVI* were used in this study. Their calculation formulas are as follows:

(13) $$NDVI=\frac{\rho_{nir}-\rho_{red}}{\rho_{nir}+\rho_{red}}$$

(14) $$RDVI=\frac{\rho_{nir}-\rho_{red}}{\sqrt{\rho_{nir}+\rho_{red}}}$$

(15) $$SAVI=\frac{\left(1+0.5)*(\rho_{nir}-\rho_{red}\right)}{\rho_{nir}-\rho_{red}+0.5}$$

(16) $$TCARI=3\left[\left(\rho_{rededge}-\rho_{red})-0.2(\rho_{rededge}-\rho_{green})*(\rho_{rededge}/\rho_{red}\right)\right]$$

where $\rho_{nir}$, $\rho_{red}$, $\rho_{rededge}$, and $\rho_{green}$ are the reflectance values of ground objects in the near-infrared, red, red-edge, and green bands. For statistical analysis, the R programming language (R-3.4.3, https://www.r-project.org/) and the lm() function were used. The coefficient of determination (R^2^) and root-mean-square error (RMSE) were used as evaluating indicators.

## 3. Results

### 3.1. NDVI and the Fraction of Vegetation Cover of Maize

**Figure 3:**
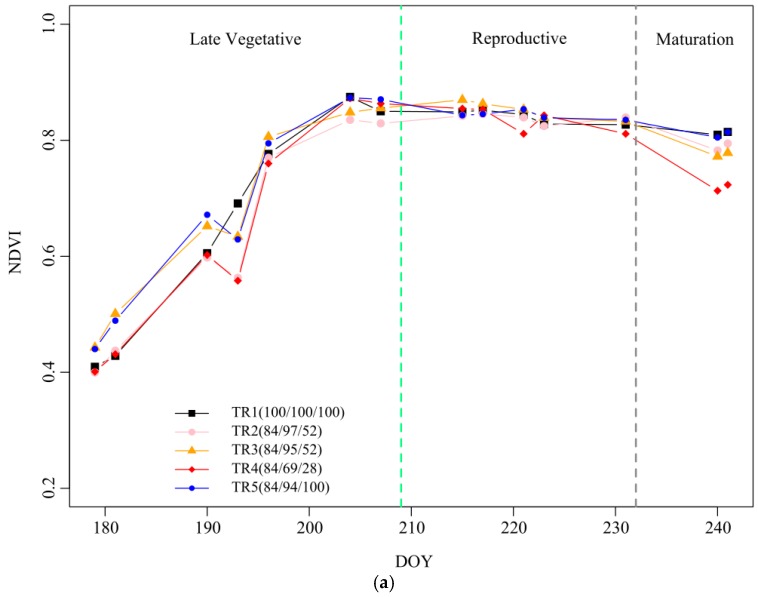

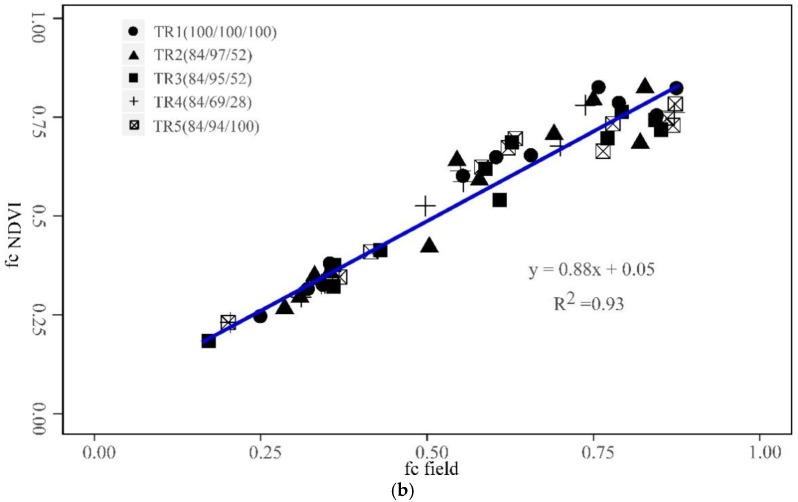
(**a**) Measured normalized difference vegetation index (*NDVI*) values under different levels of deficit irrigation in 2017. The black, pink, orange, red, and blue solid lines represent TRs 1–5, respectively. The green dotted line is the boundary between the late vegetation and reproductive stages. The gray dotted line is the boundary between the reproductive and maturation stages. (**b**) Relationship between the fraction of vegetation cover (*f_c_*) calculated from *NDVI* values and *f_c_* based on field measurements derived from the sampling plots.

It was observed that the *NDVI* values under different levels of deficit irrigation did not significant differ in the reproductive stage but changed significantly in the maturation stage, with a decrease of 0.09 in TR4 compared to TR1. The results of *f_c_* calculated from the *NDVI* (*f_c NDVI_*; Equation (5)) were compared with *f_c_* based on field measurement (*f*_c field_) for the maize (Figure 3b). Both *f_c_* values showed good agreement for all treatments, with an R^2^ value of 0.93 and regression coefficient b close to 1.0.

### 3.2. The K_c_ of Maize

**Figure 4:**
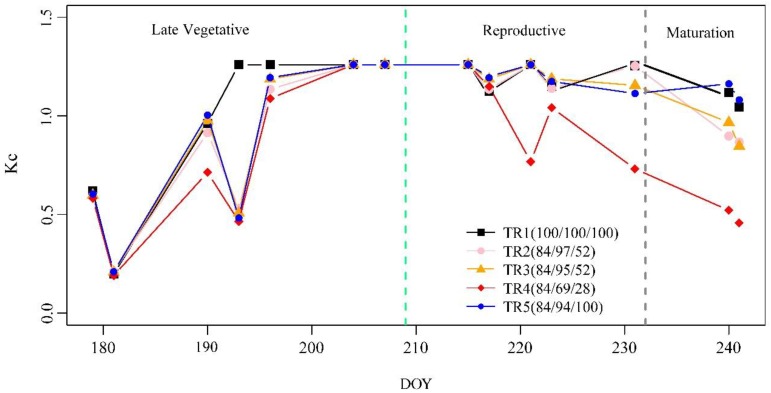
*K_c_* change curves for each deficit irrigation treatment during 2017.06.26~2017.08.29. The black, pink, orange, red, and blue solid lines represent TRs 1–5, respectively. The green dotted line is the boundary between the late vegetation and reproductive stages. The gray dotted line is the boundary between the reproductive and maturation stages.

Finally, the *K_c_* values were obtained by using the soil water balance model in the sampling plots. Figure 4 depicts the daily changes in *K_c_* for each deficit irrigation treatment during 2017.06.26~2017.08.29. *K_c_* increased after irrigation/rainfall, reaching a maximum around DOY 205, and then slowly decreased, responding well to irrigation/rainfall events. The *K_c_* values for the different levels of deficit irrigation treatments in the late vegetative, reproductive, and maturation stages had a clear numerical gradient. For example, the average *K_c_* values in the late vegetative stage were low, while the average *K_c_* values in the reproductive stage were maintained at a high level, and the average *K_c_* values in the maturation stage slowly decreased. In addition, the *K_c_* values for the different levels of deficit irrigation treatments were significantly different. For example, compared with TR1 in the reproductive and maturation stages, the *K_c_* value was significantly decreased for TR4 in the reproductive and maturation stages.

### 3.3. Estimation of K_c_ Using Two Different Methods

The results of *K_c_* estimated using the model in Equation (11) (*K_c_*-1) were compared with those from the model in Equation (12) (*K_c_*-2) for the maize under different treatments (Table 3). The *K_c_* values derived via the two different methods showed a good fit, and their coefficients of determination R^2^ varied from 0.68 to 0.80. The RMSE values were small, within the range of 0.140 to 0.322, indicating adequate stability and fairly tight dispersion in the datasets. However, when the water stress was more serious, both the R^2^ and RMSE were decreased. For example, for *K_c_*-1 with 32% water applied difference between TR1 and TR4, the R^2^ and RMSE values were 0.80 and 0.140 and 0.68 and 0.232, respectively. Compared with the *K_c_*-2 values, the *K_c_*-1 values had better performance as determined by the R^2^ and lower RMSE in all treatments. Therefore, the *K_c_*-1 model was chosen to establish the relationship between the VIs and *K_c_*.

### 3.4. Crop Coefficient Maps Based on UAV Multispectral Remote Sensing Imagery

**Figure 5:**
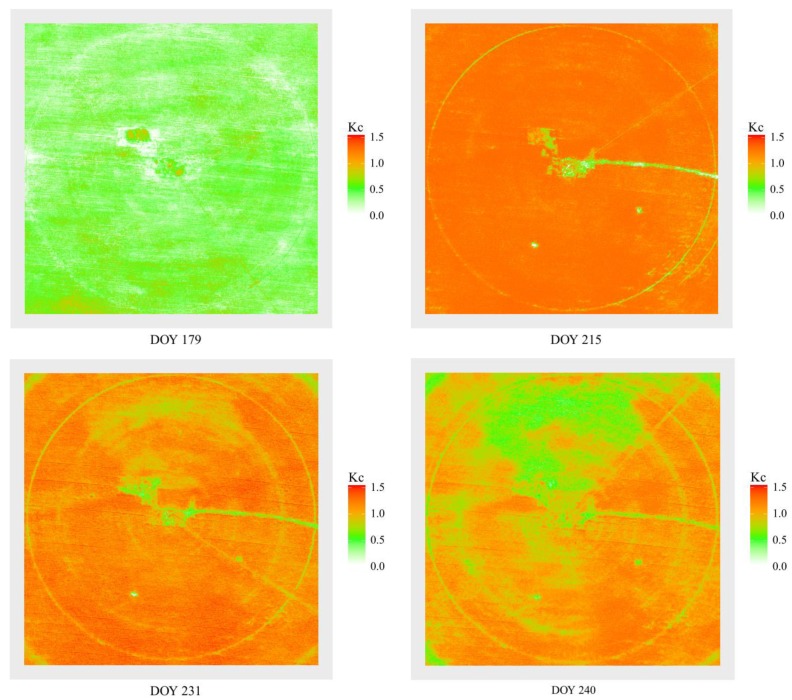
Maize crop coefficient maps retrieved using *K_c_* vs. VI regression models (Equation (11)) derived from unmanned aerial vehicle (UAV) multispectral imagery for DOY 179, 215, 231, and 240 in 2017.

Equation (11) was used to retrieve maize crop coefficient maps (Figure 5) based on UAV multispectral remote sensing imagery for DOY 179, 215, 231, and 240. The *K_c_* values of maize under each irrigation treatment showed no significant differences at DOY 179 and DOY 215. In addition, the *K_c_* status of maize under each irrigation treatment showed spatial variations at DOY 231 and DOY 240.

## 4. Discussion

UAV multispectral technology has been widely used in precision agriculture, but there are also some challenges that need to be solved in the rapid, accurate, and economical estimation of crop coefficients [32,53,54]. Previous studies have observed a high correlation between crop coefficients and VIs obtained from multispectral images, especially *NDVI* [23,55,56,57]. For example, Mutiibwa and Irmak [58] qualified the effectiveness of using AVHRR-*NDVI* data to estimate *K_c_* based on a regression model for the U.S. High Plains and showed a good prediction accuracy with an R^2^ value of 0.72 and an RMSE of 0.12. In another study, Kamble et al. [59] derived *K_c_* values from MODIS-*NDVI* data using a simple linear regression model, resulting in an R^2^ of 0.91 and an RMSE of 0.16.

Previous studies have also reported *NDVI*-based *K_c_* being successfully applied in many crops, such as maize [60,61,62,63], wheat [15,64], olive orchards [63,65], barley [63,66], sunflower [64], etc. For example, Pôças et al. [64] proposed a combined approach based on *NDVI* for maize, barley, and olive orchards and showed adequate results for supporting irrigation management. Calera et al. [33] and Cuesta et al. [66] validated *NDVI*-based *K_c_* values based on a regression model in Castilla La Mancha regions for barley and sunflower irrigated using sprinklers.

However, most studies have established the relationship between *K_c_* and VIs for nonstressed conditions or for conditions of a dry soil surface, which cannot appropriately depict the actual conditions of crop management [6]. Crop coefficients derived from VIs often do not consider the abovementioned *K_s_*, which should be used to obtain the actual *K_c_* under water or salinity stress [7,21]. Stagakis et al. [67] found that most optical indices such as *NDVI* are suitable for tracking the effects of long-term water stress on crops, while they are not useful as indicators to detect and monitor early water stress conditions. In this work, we also found that the *NDVI* values under different levels of deficit irrigation did not significantly differ in the reproductive stage, but they changed significantly in the maturation stage, with a decrease of 0.09 in TR4 compared to TR1 (Figure 3a). This is because crops may prevent damage through photo-protection strategies to reduce the leaf absorbance and reflectance changes during short-term water stress [68]. Moreover, crops consume extra energy by reducing chlorophyll b and interconverting xanthophyll cycle pigments [69]. Therefore, previous studies found that VIs are prone to reflecting the chlorophyll and xanthophyll content, which are commonly used to monitor crop water stress status. For instance, Baluja et al. [70] assessed vineyard water status by *TCARI*/OSAVI with R^2^ values of 0.58 and 0.84 (*n* = 10) when compared to stem water potential and stomatal conductance, respectively. Here, the *CWSI* based on regression models was used to obtain *K_s_* from UAV multispectral orthomosaic images, and two *CWSI* estimation methods were derived from *TCARI*/*RDVI* and *TCARI*/*SAVI*. These two indices were designed to detect crop water stress status in a more robust and adequate manner. In a relevant study by Zhang et al. [19], *TCARI*/*RDVI* and *TCARI*/*SAVI* were used to evaluate the water stress status of maize under different levels of deficit irrigation, with respective R^2^ values of 0.81 and 0.80 at the late reproductive and maturation stages.

The *f_c_* calculated by *NDVI* and the *f_c_* based on field measurement were compared. It was clear that the *f_c_* calculated by *NDVI* had a high correlation (R^2^ = 0.93) with the field measurement. Previous studies have shown that the relationship between *f_c_* and *NDVI* shows good agreement [49,51]. Considering the relationship between *f_c_* and *K_e_*_,_ we established the *K_e_* regression model. Finally, we obtained two different models for *K_c_* estimation. Both approaches to *K_c_* in all growth periods agreed with the measured *K_c_* data, with R^2^ and RMSE values varying from 0.68 to 0.80 and from 0.140 to 0.322, respectively (Table 3). Compared to *TCARI*/*SAVI*, *TCARI*/*RDVI* can more accurately characterize maize conditions in each irrigation treatment, with smaller estimated deviations. These results are likely due to the lower sensitivity of *TCARI*/*SAVI* to the influence of different water treatments. Therefore, we chose *TCARI*/*RDVI* to establish models between VIs and *K_c_*. When the water stress conditions were more serious, the *K_c_* model based on *TCARI*/*RDVI* was less accurate. For example, the R^2^ and RMSE values of the *K_c_* model in TR1 were 080 and 0.140, while the R^2^ and RMSE values of the *K_c_* model in TR4 were 0.68 and 0.232, with 32% water applied difference. The reason for this phenomenon may be that optical VIs do not allow the precise detection of serious water stress [65]. Similar phenomena were also found by Zhang and Zhou [29], Espinoza et al. [71], and Zulini et al. [72].

**Figure 6:**
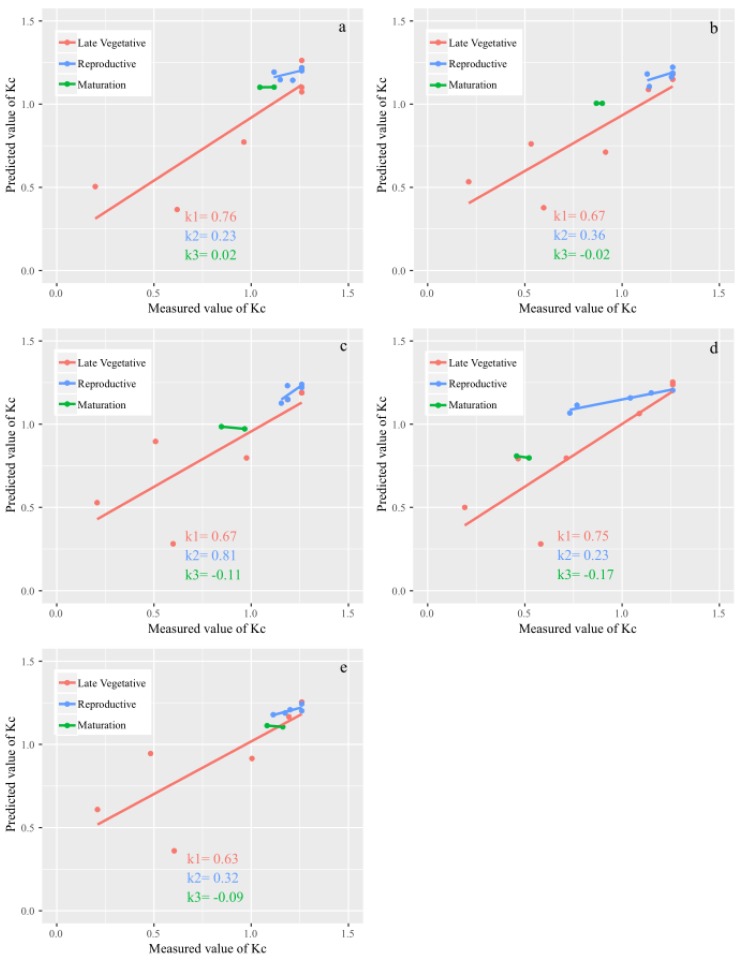
The relationship between the measured *K_c_* and predicted *K_c_* retrieved by VI regression models (Equation (11)) under different water treatments in the late vegetative stage, reproductive stage, and maturation stage. (**a**–**e**) represent TRs 1–5, respectively.

The relationship between the measured *K_c_* and predicted *K_c_* based on vegetation indices under different water treatments was compared in three different growth stages. There was a rapidly decreasing trend in the slopes of the linear regression models between the measured *K_c_* and the predicted *K_c_* throughout the growth phase (Figure 6), indicating that the correlation of the measured *K_c_* with the predicted *K_c_* obtained from vegetation indices in the late vegetative stage was higher than those in the two other growth stages. It could be also observed that when water stress was more serious in the maturation stage, a higher slope value was found, such as −0.17 in TR4. The results showed that UAV multispectral VIs could distinguish different levels of deficit irrigation treatments. Overall, multispectral VIs (*NDVI* and *TCARI*/*RDVI*) could be used to monitor the *K_c_* of field maize during the whole growth period and under different water treatments.

From the *K_c_* maps retrieved by *TCARI*/*RDVI* and *NDVI*, we found that the *K_c_* values of maize in each irrigation treatment did not significantly differ at DOY 179 and DOY 215. The retrieved *K_c_* values reflected an initial nonstress situation and water supply conditions during the late vegetative stage, respectively. However, the *K_c_* status of maize in each irrigation treatment showed spatial variations at DOY 231 and DOY 240. Compared to the *K_c_* values calculated from on-site measurements, the *K_c_* values based on the VI regression models could better reflect the management conditions of maize at the field scale. These results indicated that the average *K_c_* based on VIs was more reasonable due to it considering the entire treatment region.

## 5. Conclusions

Information obtained from the remote sensing of UAV multispectral images can be applied to irrigation water management in farm-scale areas. In the present study, the main objective was to test the suitability of estimating the *K_c_* based on VIs compared to on-site measured values for maize under different levels of deficit irrigation treatments at the farm scale. Our results confirmed that *f_c_* values derived from the *NDVI* equation had a good correlation with *f_c_* values based on field observations, with R^2^ = 0.93. Compared to that using *TCARI*/*SAVI*, the *K_s_* retrieved using *TCARI*/*RDVI* better reflected the actual *K_c_*, with R^2^ = 0.68–0.80 and RMSE = 0.140–0.232. In summary, this study demonstrated that UAV-based multispectral images can be used to map the maize crop coefficient *K_c_* and monitor irrigation requirements at the farm scale with a high temporal and spatial representation. Nevertheless, further studies are desirable to better test the methodology for other crops, and multispectral images can be combined with data from other sensors mounted on UAVs to provide more information about water status, particularly thermal cameras.

## Figures and Tables

**Table 1:** Experimental treatments and total applied water depth (percentage of full irrigation treatment in parentheses) that includes the amount of irrigation and precipitation in the late vegetative, reproductive, and maturation stages in 2017 (taken from Zhang et al. [19]).

Treatment	Applied Water Depth/mm			
	Late Vegetative (06.20–07.28)	Reproductive (07.29–08.20)	Maturation (08.21–09.07)	Total
TR 1	188 (100%)	132 (100%)	82 (100%)	402
TR 2	158 (84%)	128 (97%)	43 (52%)	329
TR 3	158 (84%)	125 (95%)	43 (52%)	326
TR 4	158 (84%)	91 (69%)	23 (28%)	272
TR 5	158 (84%)	124 (94%)	82 (100%)	365

**Table 2:** The main mean meteorological data during the study period, including the late vegetative stage, reproductive stage, and maturation stage in 2017.

Parameter	Late Vegetative (06.26–07.28)	Reproductive (07.29–08.20)	Maturation (08.21–29)
Mean air temp./°C	24.33	22.11	17.21
Max air temp./°C	37.30	31.31	25.46
Min air temp./°C	11.70	13.61	9.24
Min relative humidity/%	19.41	29.78	33.23
Mean net solar radiation/MJ·m^−2^·day^−1^	13.08	10.98	3.00
Mean wind speed/m·s^−1^	0.66	0.47	0.28
Rainfall/mm	2.8	38.8	2.8

**Table 3:** Coefficient of determination (R^2^) and root-mean-square error (RMSE) values from two different predictions of *K_c_*. Values were calculated using Equation (11) (*K_c_*-1) and Equation (12) (*K_c_*-2).

Treatment	*K_c_*-1		*K_c_*-2	
	R^2^ (*n* = 14)	RMSE	R^2^ (*n* = 14)	RMSE
TR 1	0.80 ***	0.140	0.79 ***	0.177
TR 2	0.78 ***	0.150	0.79 ***	0.221
TR 3	0.71 ***	0.174	0.72 ***	0.223
TR 4	0.68 ***	0.232	0.73 ***	0.322
TR 5	0.70 ***	0.180	0.70 ***	0.241

*** *p* < 0.001.
